# Raman Spectroscopy Detects Changes in Carotenoids on the Surface of Watermelon Fruits During Maturation

**DOI:** 10.3389/fpls.2022.832522

**Published:** 2022-05-31

**Authors:** Tushar Dhanani, Tianyi Dou, Kishan Biradar, John Jifon, Dmitry Kurouski, Bhimanagouda S. Patil

**Affiliations:** ^1^Vegetable and Fruit Improvement Center, Department of Horticultural Sciences, College Station, TX, United States; ^2^Department of Biochemistry and Biophysics, Texas A&M University, College Station, TX, United States; ^3^Department of Biochemistry, Texas A&M University, College Station, TX, United States; ^4^Texas A&M AgriLife Research, Weslaco, TX, United States

**Keywords:** Raman spectroscopy, non-invasive, ripeness, carotenoids, external quality

## Abstract

A non-invasive and non-destructive technique, Raman spectroscopy, was explored to distinguish different maturity stages (20, 30, 40, and 50 days after anthesis) of watermelon (*Citrullus lanatus*) fruits from four cultivars: Fascination, Orange Crisp, Amarillo and Crimson Sweet. Spectral acquisition from the fruit surface was carried out at the wavelength range of 400–2,000 cm^−1^ using a handheld Raman spectrometer equipped with 830 nm laser excitation source. The spectra were normalized at 1,438 cm^−1^ which was assigned to CH_2_ and CH_3_ vibration. Detecting changes in the spectral features of carotenoids on the surface of watermelon fruits can be used as a marker to monitor the maturity of the fruit. The spectral analysis confirmed the presence of two major carotenoids, lutein and β-carotene, and their intensity decreased upon maturity on the fruit surface. Identification of these pigments was further confirmed by resonance Raman spectra and high-performance liquid chromatography analysis. Results of partial least square discriminant analysis of pre-processed spectra have demonstrated that the method can successfully predict the maturity of watermelon samples with more than 85% accuracy. Analysis of Variance of individual Raman bands has revealed a significant difference among the stages as the level of carotenoids was declined during the ripening of the fruits. Thus, Raman spectral signatures can be used as a versatile tool for the non-invasive determination of carotenoid changes on the watermelon fruits’ surface during ripening, thereby enabling effective monitoring of nutritional quality and maturity indices before harvesting the watermelon.

## Introduction

The maturity at harvest significantly affects the quality of fruits and vegetables and the postharvest value chain (Erkan and Dogan, 2019). Non-climacteric fruits such as watermelon (*Citrullus lanatus*) only reach ideal quality for consumption when allowed to ripen on the parent plant (Paul et al., 2012). Usually, the maturity of watermelon fruits is assessed based on ground spot yellowness, loss of shine, thumping, or the number of senescent tendrils. However, these indicators are highly variable and do not apply uniformly to all genotypes. Furthermore, it is challenging to predict the maturity from rind color pattern, as no color breaks is visible as ripening proceeds (Vinson et al., 2010). It is, therefore, crucial to choose a suitable harvest time for proper postharvest management of watermelon. Non-destructive techniques for appropriate pre- and postharvest handling have received much attention by replacing time-consuming and labor-intensive conventional techniques for monitoring the quality of fruits (Arendse et al., 2021). Several non-destructive methods such as acoustic impulse response (Stone et al., 1996; Diezma-Iglesias et al., 2004; Ke et al., 2009; Zhang et al., 2010; Pintor et al., 2016), dielectric spectroscopy (Nelson et al., 2007a,b), laser Doppler vibrometry (Abbaszadeh et al., 2011a,b, 2013a,b, 2014, 2015a,b), machine vision systems (Ali et al., 2017; Jie and Wei, 2018), surface elastic waves (Stone et al., 1996; Ikeda et al., 2015; Ali et al., 2017), near-infrared and visible spectroscopy (Flores et al., 2008; Jie et al., 2014, 2019) have been studied for internal and external evaluation of watermelon quality.

Among non-destructive techniques, Raman spectroscopy has received increased interest as a promising non-invasive, label-free, and field-based high-throughput phenotyping platform for precision agriculture (Akpolat et al., 2020; Payne and Kurouski, 2020). Raman spectroscopy, which emerged from the discovery of the Raman effect by C. V. Raman in 1928, is a powerful technique that detects characteristic rotational/vibrational energy levels of a molecule. Raman spectroscopy gives spectral fingerprint of the molecules, and the intensity of Raman peak is directly proportional to the molecule’s concertation (Hata et al., 2000). Development of portable Raman instruments has enabled rapid in-field measurement of chemical fingerprints and phenotyping of different plant properties (Conrad and Bonello, 2015). Raman spectroscopy has been extensively applied for structural analysis, quality and safety control, classification, and quantification of fruits and vegetables such as apple, avocado, apricot, cabbage, carrot, citrus, cucumber, grape, kiwifruit, mango, citrus, olive, pear, pepper, potato, spinach, and tomato (Liu et al., 2013; Lee and Herrman, 2016; Qin et al., 2019). However, few studies have been examined the internal or external quality attributes of watermelon. Furthermore, the shorter penetration depth of source radiation makes it challenging to study internal quality attributes of fruits with a thick rind, such as watermelon. Regardless, the Raman spectrum from the surface of the watermelon could be used for quality evaluation (Arendse et al., 2018).

The present investigation applied Raman spectroscopy to study the variation of carotenoids in the rind of watermelon fruit during ripening. Carotenoids are common plant pigments and antioxidants with many beneficial properties to plant and human health. Because the Raman spectra of carotenoids vary with minor structural differences, the spectra have been widely used to identify and characterize carotenoids in biological systems. Resonance Raman spectra were acquired from pure compounds and carotenoid-rich fractions to confirm the presence of individual carotenoids and the results of Raman spectroscopy were compared with those obtained from HPLC analysis. Lastly, partial least squares-discriminant analysis (PLS-DA) was used to couple Raman spectra to the developmental stage, allowing us to predict different development stages of four watermelon cultivars. PLS-DA has been demonstrated to be a more suitable discriminant method comparing to methods like Fisher’s linear discriminant analysis or principal component-linear discriminant analysis (Yang and Yang, 2003; Lee et al., 2018).

## Materials and Methods

### Watermelon Samples and Analysis

Raman experiments were conducted using four watermelon varieties: (a) Fascination, (b) Orange Crisp., (c) Amarillo, and (d) Crimson Sweet. Watermelon fruits were harvested at 20 days (Stage A), 30 days (Stage B), 40 days (Stage C), and 50 days (Stage D) after anthesis. Fruits were harvested in the Texas A&M University experimental fields located at the Horticultural Research and Extension Facility near Snook, TX during the harvest season in the year 2020. Fruits were directly transported to the laboratory and washed with water before analysis. Reference compounds lutein, β-carotene and lycopene, reagent-grade acetone, chloroform, HPLC grade methanol, and *tert*-butyl methyl ether were purchased from Sigma-Aldrich, United States. Nanopure water (Barnstead/Thermolyne, Dubuque, IA, United States) was used for HPLC analysis.

### Raman Spectrum Acquisition and Data Processing

Three fruit samples at each stage per variety were used for Raman spectrum acquisition. Spectra ranging from the stalk end to the flower end of each fruit’s surface were acquired using a hand-held spectrometer (Resolve, Agilent, United States) equipped with a 495 mW laser source with 830 nm excitation wavelength. Spectral acquisition time was set at 1 s. Spectra were acquired using the surface mode setting built into the portable instrument. The barrier scan mode was used, but due to different thicknesses of watermelon rinds, the parameters of the barrier scan varied from scan to scan. Before multivariate analysis, pre-processing such as area normalization, mean centering, a Kruskal–Wallis test was carried out using MATLAB 2020a software. All spectra were normalized to 1,439 cm^−1^ bands, corresponding to CH_2_ and CH_3_ vibration, which cannot be assigned to any specific class of biomolecule. Kruskal–Wallis one-way analysis tests if the median in a set of samples is significantly different from other classes in the set. The null hypothesis for Kruskal–Wallis test is that there is no significant difference in the band of interest. The significance level is 0.05. The results report a 95% CI for the true value of median for each compared group. The multi compare function was used to overlap the confidence intervals. Partial least squares discriminant analysis (PLS-DA) was conducted using MATLAB PLS_Toolbox 8.6.2.

#### Resonance Raman Study

Resonance Raman spectra of individual carotenoids (pure standards dissolved in extraction solvent) and carotenoid-rich fractions (extracted as described below) were acquired using a confocal inverted microscope (Nikon, Model TE-2000U) with 20x dry Nikon objective (NA = 0.45). A solid-state laser (Necsel SLM785.0-FS-01) was used for 485 nm excitation. Lutein, lycopene, and beta-carotene were dissolved in extraction solvent (acetone: chloroform, 7:3), and spectra were collected from solutions individually. The signal was collected in a backscattering geometry and sent to a spectrometer (Princeton Instruments, IsoPlane-320) equipped with a 600 groove/mm grating. Prior to entering the spectrograph, the Rayleigh scattering was filtered with a long-pass filter (Semrock, LP03-785RS-25). The dispersed light was then sent to the CCD (PIX-400BR). All data were processed using GRAMS/AI 7.0 (Thermo Galactic, Salem, NH). Spectra were baselined using multiple-point baseline correction in GRAMS/AI 7.0 (Thermo Galactic, Salem, NH).

#### Extraction of Carotenoids

After the spectral acquisition, the watermelon rind was separated from the flesh and blended using a laboratory blender (Magic Bullet). Five grams of crushed rind was extracted in dark using extraction solvent (acetone: chloroform, 7:3), vortexed for 1 min at 1814 x *g*, homogenized (850 Homogenizer, Fisher Scientific, Waltham, Massachusetts, United States) and sonicated (Cole-Parmer Ultrasonic cleaner 8,893) in ice-cold water for 30 min. Sample tubes were centrifuged (Beckman Model TJ-6, Ramsey, Minnesota, United States) at 4480 x *g* for 10 min. The lower organic layer was collected in another tube and the extraction procedure repeated on the sample to ensure the maximum recovery of analytes. Organic layers from two extractions were pooled together, a 5-ml aliquot was transferred to amber glass vial, and solvent was removed under vacuum at room temperature. The residue was redissolved in 1 ml of extraction solvent. Carotenoid-rich fractions were stored at −80°C until further analysis.

#### HPLC Profiling of Carotenoids

A Waters 1525 HPLC system (Milford, MA, United States) equipped with 2996 PDA detector, a 717 Plus autosampler was used for quantification. Separation of carotenoids was achieved on YMC carotenoid C_30_ (250 mm × 4.6 mm) column. The mobile phase constitutes a mixture of (A) *tert*-butyl methyl ether (TBME):methanol:water (85:13:2), and (B) methanol:TBME:water (85:13:2). The gradient was programmed as follows: 0–3 min, 85% (B); then 35%, 33%, 20%, 10%, and 85% B at 6, 12, 19, 23 and 25 min; after that, the initial condition was restored for 3 min. For analysis, 20 μl of each aliquot was injected and the chromatogram was monitored between 210 and 700 nm. Statistical analysis of HPLC results was performed using Microsoft Excel 2019.

## Results and Discussion

### Identification of Carotenoids Using Raman Techniques

**Figure 1:**
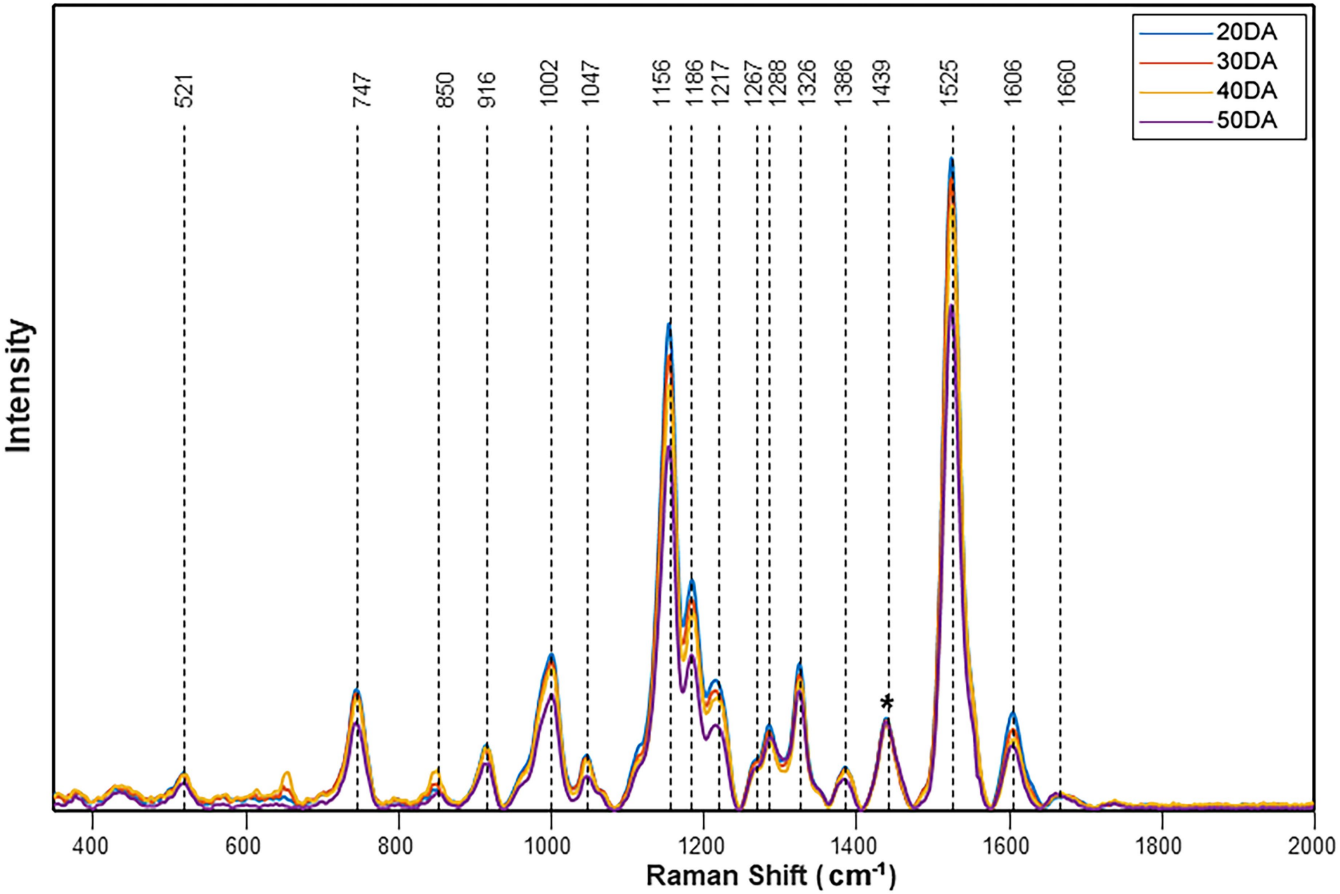
Raman spectra obtained from the surface of the watermelon fruit Fascination cultivar. The stacked spectra represent an average of 20DA = 43, 30DA = 42, 40DA = 42, and 50DA = 24. The spectra were normalized at 1,439 cm^−1^ [Marked with an asterisk (*)].

Raman spectra collected from the surface of four different cultivars of watermelon demonstrated similar profiles. The spectral feature of Fascination type watermelon at four different stages of maturity is shown in Figure 1. The bands observed at 1,002, 1,156, 1,186, 1,217, and 1,525 cm^−1^ can be assigned to carotenoids. Carotenoids show two strong Raman bands (1,525 and 1,156 cm^−1^) due to in-phase υ(C–C) and υ(C–C) stretching vibrations of the polyene chain (Qin et al., 2011). For instance, β-carotene with 11 conjugated double bonds is characterized by the bands at 1,515 and 1,157 cm^−1^. Bands at 1,186 and 1,217 can be assigned to C–C stretching vibrations coupled to either C–H in-plane bending or C–CH_3_ stretching modes (Grudzinski et al., 2016). A feature of medium intensity occurs at around 1,002–1,008 cm^−1^, corresponding to the in-phase rocking modes of the CH_3_ groups attached to the polyene chain (Schulz et al., 2005; Jehlička et al., 2014). These distinct carotenoid signals also enabled the effective monitoring of four levels of maturity index in hot peppers by Raman spectroscopy (Legner et al., 2021). Raman spectroscopy was recently explored for the *in situ*, non-destructive, and rapid quantitative analysis of photosynthetic pigments, chlorophyll, and carotenoids in tea leaves (Zeng et al., 2021). Other characteristic vibrational bands observed at 520 and 1,047 cm^−1^ can be assigned to cellulose, 915 cm^−1^ to carbohydrates, 747 and 850 cm^−1^ to pectin, 1,267 and 1,606 cm^−1^ to phenylpropanoids or lignin, 1,670 cm^−1^ to protein and 1,286, 1,327, 1,386 and 1,439 cm^−1^ to CH_2_/CH_3_ vibrations of aliphatic groups (Supplementary Table S1).

### Resonance Raman

The carotenoid bands observed from the watermelon rind surface using the handheld spectrometer were further confirmed using a confocal microscope at 485 nm excitation. In resonance Raman Spectroscopy, laser excitation frequency is chosen to be close to the frequency of a sample’s electronic transition (Merlin, 1985). The resonance Raman spectra of individual carotenoids, β-carotene, lutein, and lycopene exhibited peaks at υ = ~1,527, υ = ~1,159 and υ = 1,008 cm^−1^ (Supplementary Figure S1). These peaks match the fruit surface spectra of lutein and β-carotene, confirming the presence of these two pigments in the watermelon rind.

**Figure 2:**
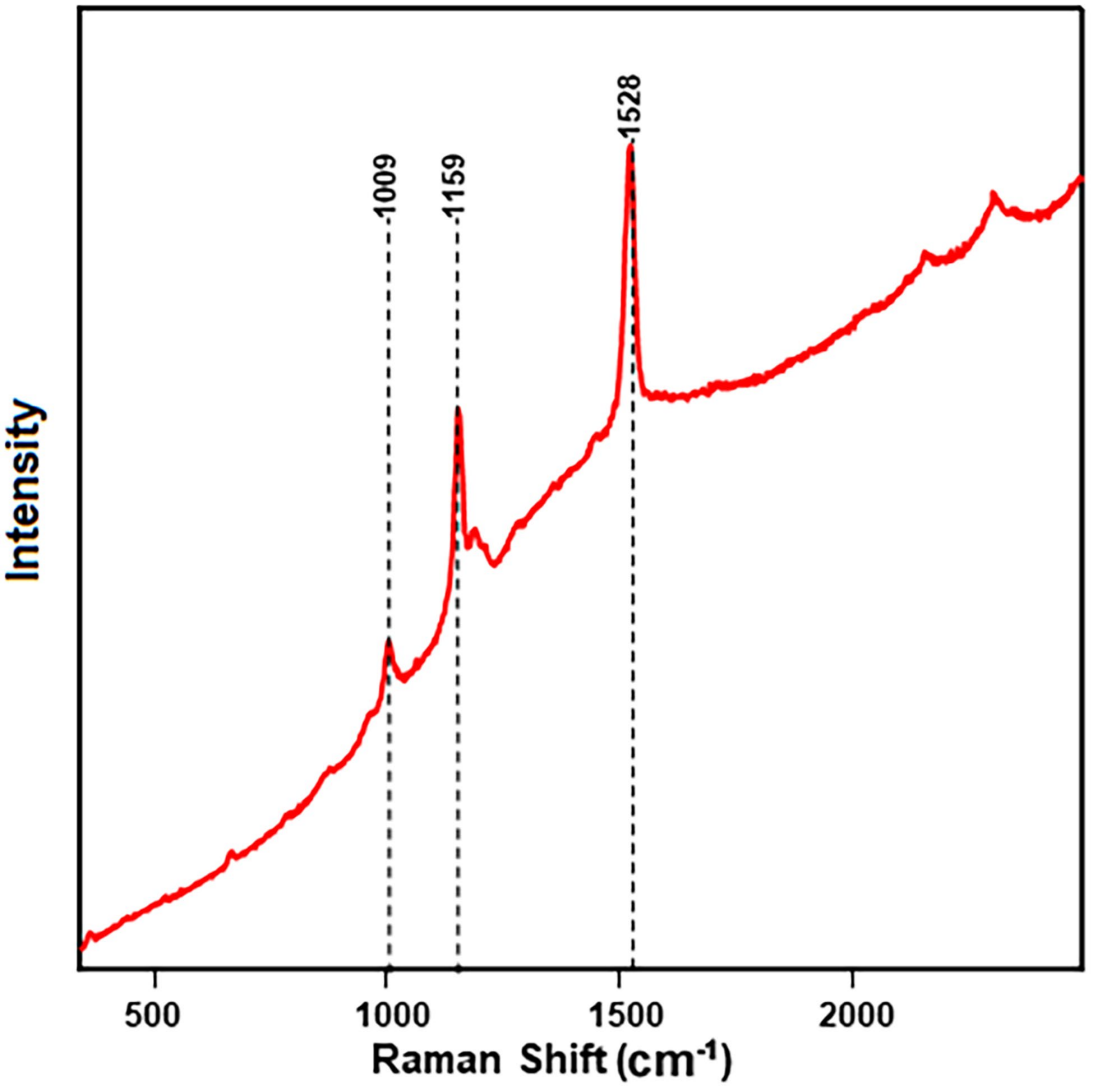
Resonance Raman spectra of carotenoid-rich fraction show prominent bands related to carotenoids obtained from the rind of the Fascination type watermelon.

However, resonance Raman spectra recorded from pure carotenoids revealed band shifting of assigned wavenumbers. Carotenoids bind biomass, which affects the main polyene chain and thus can cause a significant shift of the band position (1,008–1,002 cm^−1^) due to changes in electron delocalization. Carotenoids in different solvents can undergo slight band shifts from 1,008 (in extraction solvent) to 1,002 (in watermelon rind) cm^−1^ due to different vibronic coupling in different stages (Yu et al., 2007). Another factor affecting the band shifts is substitution at the terminal end groups of the molecule, resulting in very small wavenumber changes in the solid and liquid states. The band position of carotenoids also depends on the laser wavelength used for excitation (Jehlička et al., 2014; Harris et al., 2015). Furthermore, resonance Raman spectra of carotenoid-rich fractions of each variety at each stage confirmed the presence of lutein and β-carotene (Figure 2), further authenticated by HPLC analysis. In a previous study, a variety of intact fruits and vegetables and their juices were measured for carotenoids by resonance Raman spectroscopy and compared to concentrations determined by extraction and HPLC (Bhosale et al., 2004).

### Raman Spectral Analysis

The spectra obtained from Raman analysis were normalized at 1,438 cm^−1^, which corresponds to CH_2_ and CH_3_ bending vibrations. Since most organic compounds have these features, it is impossible to attribute these bands to a specific compound. However, normalization allows us to compare the relative intensity of the bands. The watermelon rind showed a trend of decreasing spectral intensity of the main bands from the early stage (A) to the mature stage (D). PLS-DA was conducted to determine whether watermelon maturity stages can be discriminated by using the Raman spectra. Classification results of the PLS-DA model created using Raman spectral data are presented in Table 1. The results table shows that the stage D classification, which determines the full maturity stage, was 100% accurate for three out of four cultivars, while the full maturity stage for the Fascination type watermelon was classified with 95.8% accuracy. 64.3% of spectra identified correctly as stage B and C in Fascination type watermelon, while the rest were incorrectly identified as other stages. Similar accuracy (64.3%) in prediction of stage B of the Amarillo cultivar was also observed. True prediction rates for the remaining stages ranged from 73.8% to 93% for all cultivars. Our results show that PLS-DA is able to differentiate fully matured stages with an accuracy between 95.8% and 100%. Harvesting watermelon fruits at full maturity is critical for the best teste and texture, which determines their market value.

**Table 1:** Confusion matrix computed from the PLS-DA model of Raman spectra collected from four watermelon cultivars.

Ripening stage	Total spectra	Predicted as A	Predicted as B	Predicted as C	Predicted as D	Correct (%)
**Fascination**						
A	43	**38**	5	0	0	88.4
B	42	8	**27**	7	0	64.3
C	42	2	11	**27**	2	64.3
D	24	0	0	1	**23**	95.8
**Orange crisp**						
A	43	**40**	1	2	0	93.0
B	43	0	**43**	0	0	100
C	39	4	1	**32**	2	82.1
D	40	0	0	0	**40**	100
**Amarillo**						
A	42	**42**	0	0	0	100
B	42	12	**27**	3	0	64.3
C	42	0	3	**38**	1	90.5
D	20	0	0	0	**20**	100
**Crimson sweet**						
A	42	**36**	6	0	0	85.7
B	42	9	**31**	2	0	73.8
C	42	1	4	**37**	0	88.1
D	20	0	0	0	**20**	100

Bold value is the highest number of spectrums predicted according to their ripening stage.

**Figure 3:**
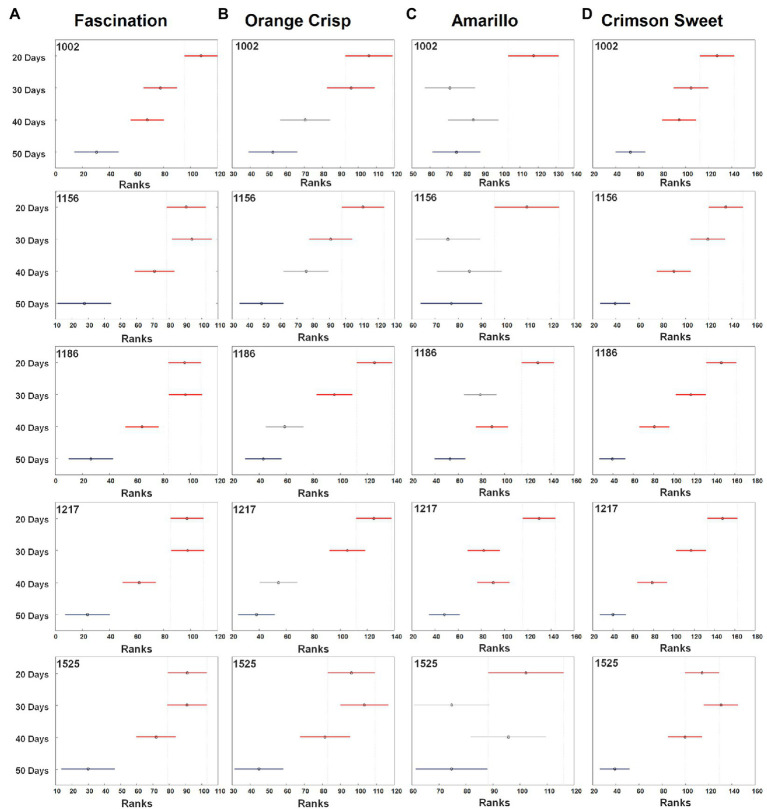
The median (circles) and 95% CI (line) for the relative intensity of bands of carotenoids observed on the surfaces of four watermelon cultivars [Fascination **(A)**, Orange Crisp **(B)**, Amarillo **(C)**, and Crimson Sweet **(D)**] at four stages of fruit development. For each stage, 20–43 spectra were collected for each cultivar. The confidence intervals were compared to 50 days stage (blue). The separation of different developmental stages is in red and unseparated is in grey. All the spectra analyzed in Kruskal–Wallis test were normalized to 1,439 cm^−1^.

The results obtained from the PLS-DA confusion matrix give an overall view of the classification model, but do not provide information about variation in the individual groups, i.e., the significant differences among maturity stages. Therefore, ANOVA was conducted to determine whether the differences in bands associated with carotenoids were statistically significant (Figure 3). In general, stage A tended to have wider confidence intervals for the true mean intensity of all the bands of carotenoids compared to the later stages (B, C and D). Despite the higher intensity in the Raman spectra, the band at 1,525 cm^−1^ could not accurately differentiate the stages of maturity for all the cultivars. For Fascination type watermelon, all the bands at stage D were significantly lower than in earlier stages. However, stages B and C did not show any significant difference in the spectral intensities. Still, they were distinguished from stages A and D. For the Orange Crisp variety, the intensity of all the studied bands at stages C and D were not significantly different from each other but had a significantly higher intensity compared to stages A and B. The band intensities in stages B and D were similar in Amarillo type watermelon. As stated earlier, all the bands at stage A were of significantly higher intensity than all other later stages. Finally, in Crimson Sweet, the intensity of bands in stages B and C were similar in Fascination type watermelon and bands in stages B, C, and D were less intense than in stage A. Despite the similarity in the intensities of certain ripening stages, the confidence interval centers for the studied varieties were observed in decreasing order except for Amarillo cultivar during maturity.

**Figure 4:**
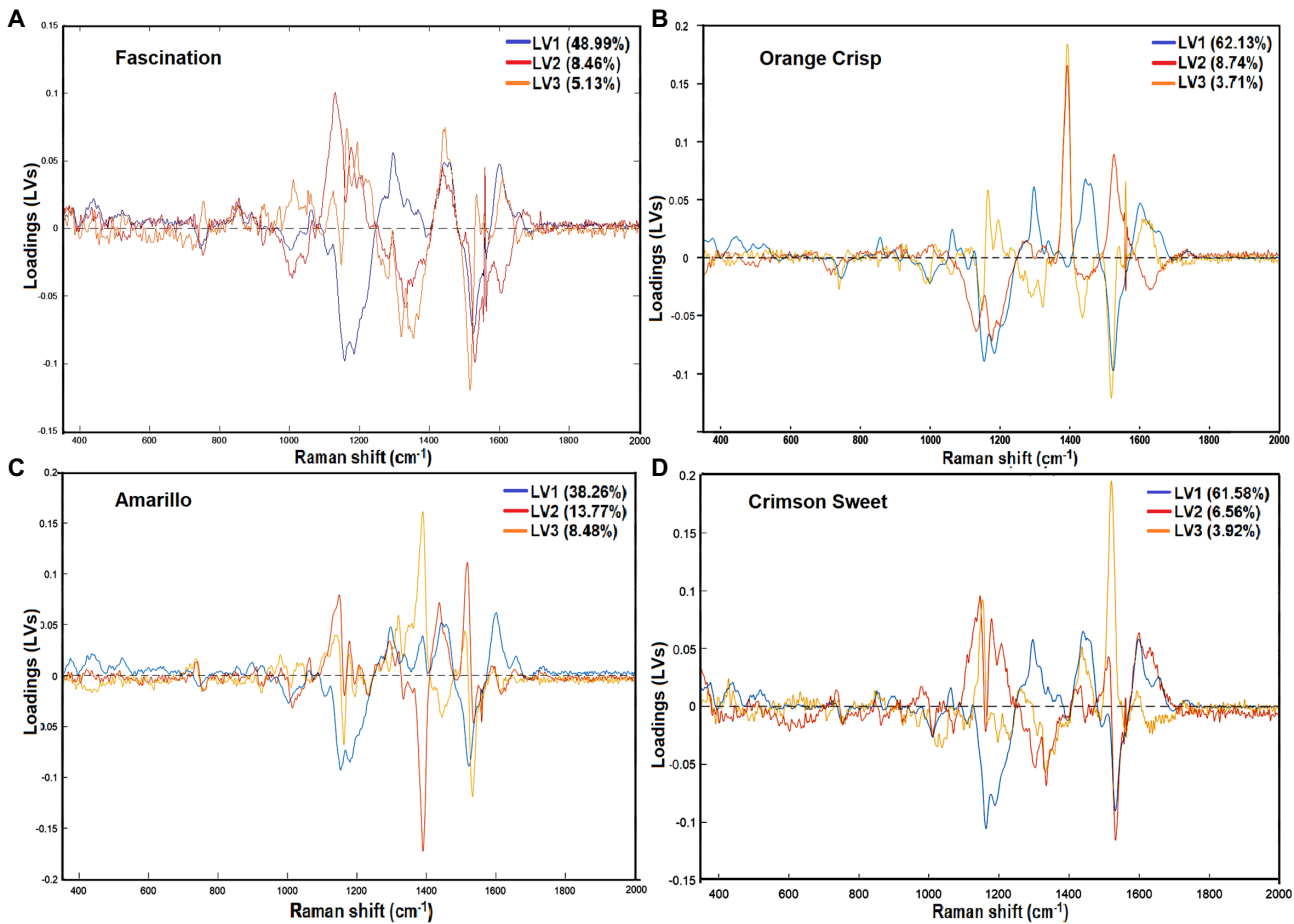
Loading plots for the first three latent variables (LVs) representing wavenumbers having the highest contribution to the maturity prediction model developed from the surface Raman spectra of four watermelon cultivars [Fascination **(A)**, Orange Crisp **(B)**, Amarillo **(C)**, and Crimson Sweet **(D)**].

The wavenumbers that were mainly accountable for classification could be observed by inspecting the loadings plot for the first three latent variables (LVs), as shown in Figure 4. Those wavenumbers are considered to be important for the differentiation between stages of maturity. Fascination cultivar’s loading plot shows that the LV with the highest contribution is at 1,156 cm^−1^ and can be assigned to the carotenoid pigments. Other variables that have the most significant contribution to LV1 correspond to regions of the Raman spectrum at 1,327 and 1,606 cm^−1^. These characteristic bands can be assigned to the chlorophylls and lignin phenylpropanoids. Investigation of the loading plot of Orange Crisp cultivar indicated that wavenumber at 1,386 and 1,525 cm^−1^ corresponds to aliphatics and carotenoids were more important for the discrimination of ripening stages. Similarly, Raman’s considerable absolute value at 1,386 and 1,525 cm^−1^ for Amarillo melons had a significant influence on classification. The Crimson Sweet cultivar’s loading plot showed that variation associated with carotenoids (1,156, 1,186, and 1,525 cm^−1^) was the most important for this discrimination. Inspection of all the cultivar loading plots showed that the band observed at 1,327 cm^−1^, which was assigned to C–H vibration of aliphatics, also had a remarkable effect on predicting maturity stages.

### HPLC Analysis

**Figure 5:**
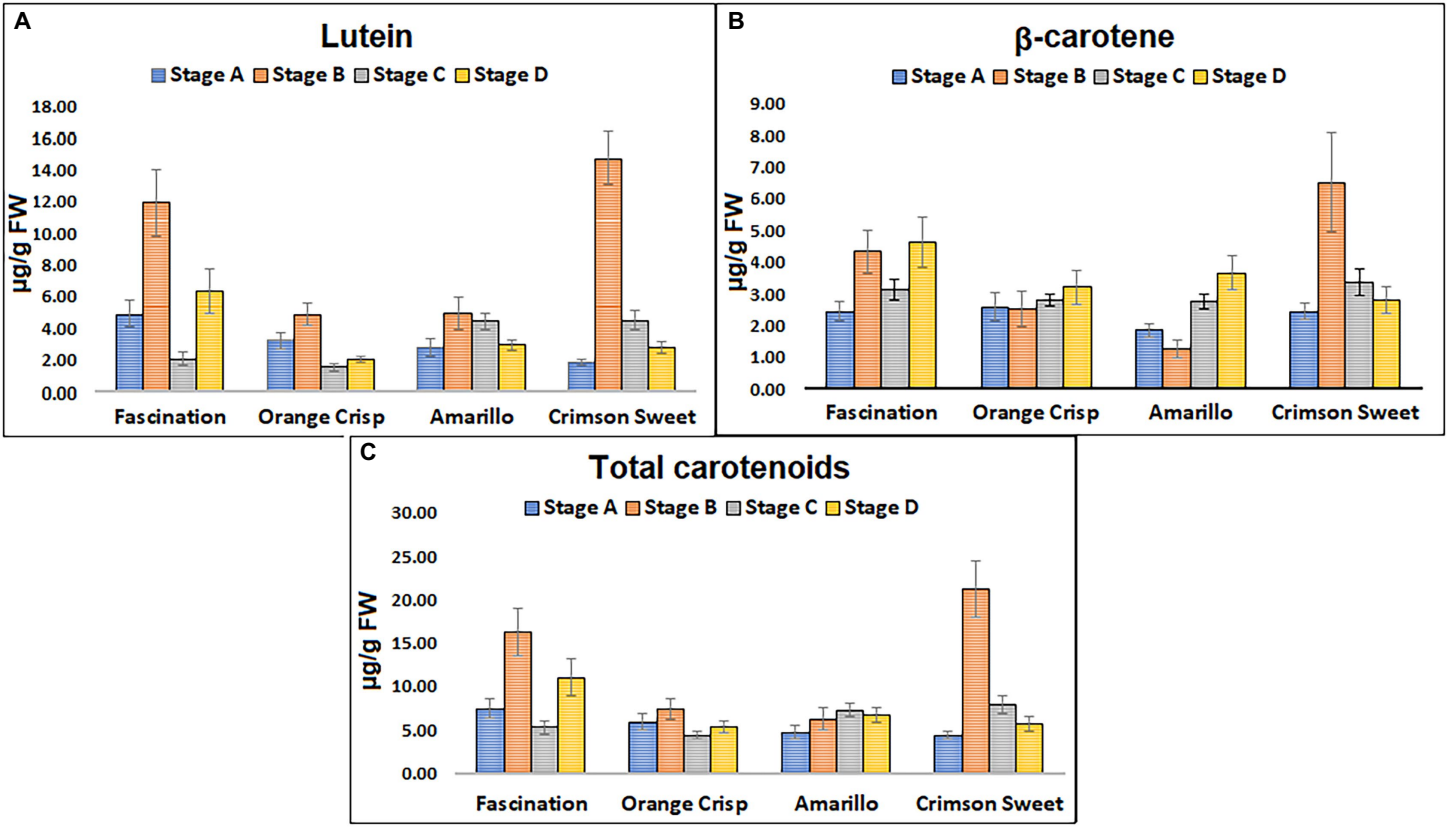
Concentration of individual pigments **(A)** lutein (μg/g FW), **(B)** β-carotene (μg/g FW), and **(C)** total carotenoids detected at different stages of ripening from the fruit rinds of four different cultivars of watermelon. Error bars represent SD from the average (*n* = 18).

An optimized HPLC method using a C_30_ RP column for the quantification of carotenoids (Supplementary Figure S2) was further used for the analysis of watermelon samples. Lutein and β-carotene were the major carotenoids identified in the watermelon rind extracts and their total concentrations ranged from 1.88 to 14.73 and 0.04 to 0.14 μg/g fresh weight (FW), respectively (Figure 5). The HPLC analysis did not confirm the presence of lycopene in the extracts congruent with the Raman experiment results. In the Fascination type watermelon, the total carotenoid level varied among ripening stages. However, the intensity of Raman bands for stage B and C were almost similar but lower than stage A and higher than stage D (Figure 3A). HPLC results show that variation in the total carotenoid level in Orange Crisp cultivar was similar to Fascination variety. ANOVA of Raman bands revealed that stages A and B had a significantly higher rank than stages C and D (Figure 3B). Significant variation in the carotenoid contents in the Amarillo type watermelon was not observed among the maturity stages. Still, an increasing trend was recorded up to stage C. The rank of carotenoid bands at stages B and D was similar from ANOVA results, and stages B to D were significantly lower than stage A (Figure 3C). The highest amount of lutein (14.73 μg/g FW) and β-carotene (6.51 μg/g FW) representing the total carotenoids (21.24 μg/g FW) was recorded in stage B of Crimson Sweet. The level of carotenoids increased from stage A to B, then later showed a decreasing trend. The general trend observed in the Kruskal–Wallis and ANOVA results for Fascination cultivar remained true for Crimson Sweet type watermelon (Figure 3D). Further, HPLC results show a decreasing trend for stages B and C for all the cultivars except Amarillo. These trends match the Raman trend analysis results if we look at the confidence interval center in ANOVA.

## Conclusion

The study confirmed that variation in the carotenoid content measured on the surface of watermelon fruits using a Raman spectrometer can be reliably used for the non-invasive detection of fruit maturity. Raman spectral features for carotenoids were consistent throughout the ripening process with diminishing intensity at full maturity. A fast and sensitive HPLC method for carotenoids was developed using a C_30_ column with a gradient consisting of TBME, methanol, and water for the validation of Raman spectral analysis. Lutein was a prominent carotenoid followed by β-carotene in the peel of all watermelon varieties. Along with HPLC, resonance Raman confirmed the presence of lutein and β-carotene as major pigments. PLS-DA successfully classified more than 85% of samples with respect to their stage of maturity. ANOVA results of five Raman bands related to carotenoids revealed a significant difference in their intensities, thereby decreasing the carotenoid level throughout the maturation process. Raman spectroscopy is a prominent technique for identifying and characterizing carotenoids in plant tissues. The Raman bands observed at 1,002, 1,156, 1,186, 1,217, and 1,525 cm^−1^ corresponding to –C–C– and –C–C– vibrations can be used as fingerprints to characterize the carotenoids. Integrating Raman spectroscopy with other non-destructive techniques such as near-infrared region measurement, Fourier transform infrared spectrophotometry, and chemometric tools could show promising results for online quality assessment of watermelon.

## Data Availability Statement

The original contributions presented in the study are included in the article/Supplementary Material, further inquiries can be directed to the corresponding authors.

## Author Contributions

TDh, JJ, KB, and BP designed the field and lab analysis experiments. TDo and DK designed RS experiment. TD carried out all analytical work. TDh and TDo conducted, collected and interpreted results, and drafted the manuscript. TDh and TDo have equal contribution. All authors contributed to the article and approved the submitted version.

## Funding

DK and BP acknowledges the Institute for Advancing Health Through Agriculture for providing financial support. BP also acknowledges SCRI-Texas Department of Agriculture Block grant 2019-SC-1920-38, USDA-SCRI-2017- 51181-26834.

## Conflict of Interest

The authors declare that the research was conducted in the absence of any commercial or financial relationships that could be construed as a potential conflict of interest.

## Publisher’s Note

All claims expressed in this article are solely those of the authors and do not necessarily represent those of their affiliated organizations, or those of the publisher, the editors and the reviewers. Any product that may be evaluated in this article, or claim that may be made by its manufacturer, is not guaranteed or endorsed by the publisher.

## Supplementary Material

The Supplementary Material for this article can be found online at: https://www.frontiersin.org/articles/10.3389/fpls.2022.832522/full#supplementary-material
