# Supplementary material for: Raman Spectroscopy Detects Changes in Carotenoids on the Surface of Watermelon Fruits During Maturation
Source: Front Plant Sci. 2022 May 31;13:832522. doi: 10.3389/fpls.2022.832522 (PMC9194672; doi:10.3389/fpls.2022.832522)
Supplement: Supplementary file 1 [file Data_Sheet_1.docx]

**Raman spectroscopy detects changes in carotenoids on the surface of watermelon fruits during maturation**

Supplementary Material

| - **TABLE S1 \|** Assignment of vibrational bands observed in the spectra collected from the four different watermelon cultivars. | | |
| --- | --- | --- |
| **Band (cm^-1^)** | **Vibrational mode** | **Assignment** |
| 520 | υ(C-O-C) glycosidic | Cellulose(Edwards et al., 1997) |
| 747 | γ(C-O-H) of COOH | Pectin(Synytsya et al., 2003) |
| 850 | (C6-C5-O5-C1-O1) | Pectin(Engelsen & Nørgaard, 1996; Szymańska-Chargot et al., 2016) |
| 915 | υ(C-O-C) in plane, symmetric | Cellulose, phenylpropanoids(Edwards et al., 1997) |
| 1002 | υ _3_(C-CH3 stretching) and phenylalanine | Carotenoids, protein(Farber et al., 2019; Kurouski et al., 2015; Schulz et al., 2005; Tschirner et al., 2009) |
| 1047 | υ(C-O) + υ(C-C) +δ(C-O-H) | Cellulose, phenylpropanoids(Edwards et al., 1997; Farber et al., 2019) |
| 1156 | C-C stretching, υ(C-O-C), υ(C-C) in glycosidic linkage, asymmetric ring breathing | Carotenoids, carbohydrates(Edwards et al., 1997; Farber et al., 2019; Schulz et al., 2005; Wiercigroch et al., 2017) |
| 1185 | υ(C-O-H) next to aromatic ring + δ(CH) | Carotenoids(Grudzinski et al., 2016), phenylpropanoids(Farber et al., 2019) |
| 1216 | δ(C-C-H) | Carotenoids, Xylan(Farber et al., 2019; Flores et al., 2008) |
| 1267 | Guaiacyl ring breathing, C-O stretching (aromatic); C=C- | Lignin(Farber et al., 2019; Grudzinski et al., 2016) |
| 1286 | δ(C-C-H) | Aliphatics(Marcia et al., 2007) |
| 1327 | δ(CH2) bending | Aliphatics, cellulose, phenylpropanoids(Edwards et al., 1997) |
| 1386 | δ(CH2) bending | Aliphatics(Marcia et al., 2007) |
| 1439 | δ(CH2) + δ(CH3) | Aliphatics(Marcia et al., 2007) |
| 1525 | -C=C- in plane | Carotenoids(Adar, 2017; Devitt et al., 2018) |
| 1606 | υ(C-C) ring + δ(CH) | Lignin, Phenylpropanoids(Agarwal, 2006; Kang et al., 2016) |
| 1670 | Amide I | Proteins(Kurouski et al., 2015) |


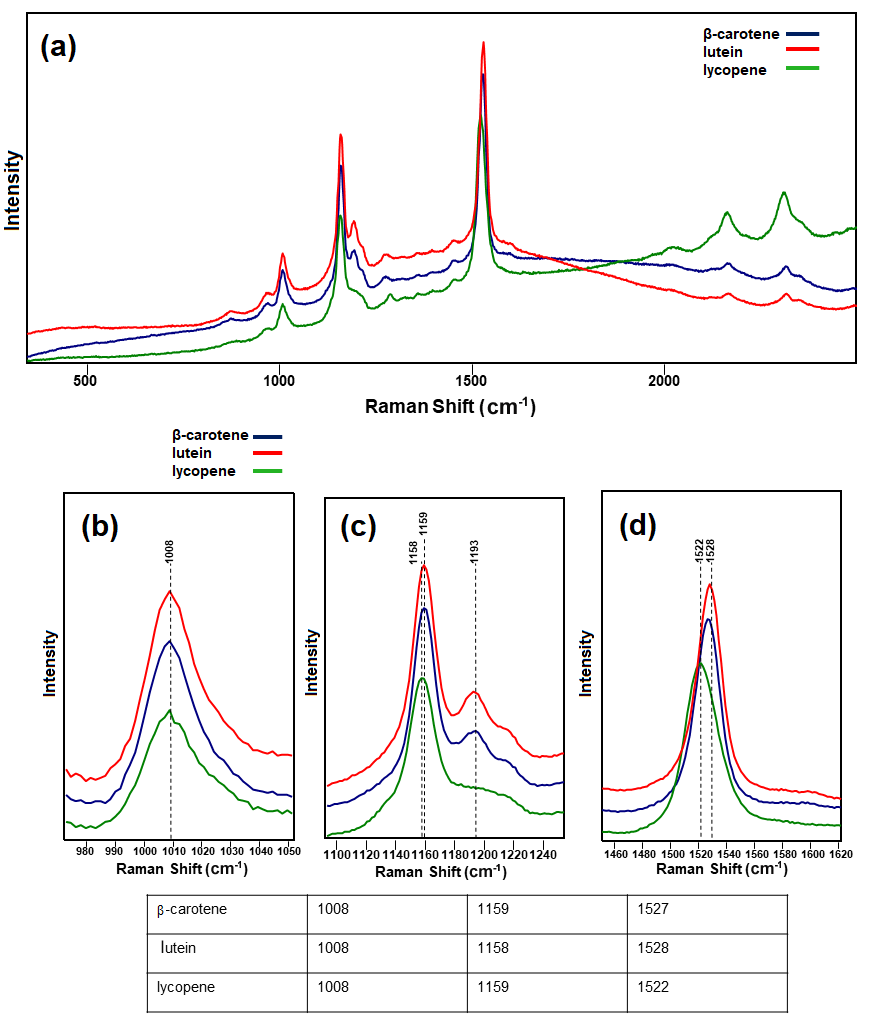


**Figure** **S1** | Resonance Raman spectra of (A) mixture of standard β-carotene, lutein and lycopene, (B), (C), (D) and table represent the band positions of the individual carotenoids.


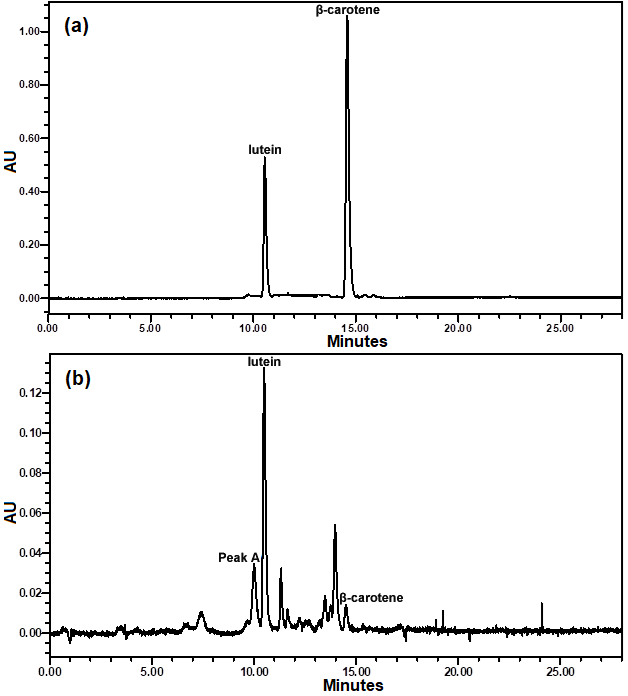


**Figure S2 |** HPLC chromatograms recorded at 450 nm of (a) standard mixture of lutein and β-carotene (b) carotenoid-rich fraction obtained from watermelon rind of Fascination cultivar.
